# Weight Loss Reversed Obesity-Induced HGF/c-Met Pathway and Basal-Like Breast Cancer Progression

**DOI:** 10.3389/fonc.2014.00175

**Published:** 2014-07-08

**Authors:** Sneha Sundaram, Trinh L. Le, Luma Essaid, Alex J. Freemerman, Megan J. Huang, Joseph A. Galanko, Kirk K. McNaughton, Katharine M. Bendt, David B. Darr, Melissa A. Troester, Liza Makowski

**Affiliations:** ^1^Department of Nutrition, Gillings School of Global Public Health and School of Medicine, The University of North Carolina at Chapel Hill, Chapel Hill, NC, USA; ^2^Lineberger Comprehensive Cancer Center, The University of North Carolina at Chapel Hill, Chapel Hill, NC, USA; ^3^Department of Cell Biology and Physiology, The University of North Carolina at Chapel Hill, Chapel Hill, NC, USA; ^4^UNC Nutrition Obesity Research Center, The University of North Carolina at Chapel Hill, Chapel Hill, NC, USA; ^5^Department of Medicine, The University of North Carolina at Chapel Hill, Chapel Hill, NC, USA; ^6^Mouse Phase I Unit, Lineberger Comprehensive Cancer Center, The University of North Carolina at Chapel Hill, Chapel Hill, NC, USA; ^7^Department of Epidemiology, The University of North Carolina at Chapel Hill, Chapel Hill, NC, USA; ^8^Department of Pathology and Laboratory Medicine, The University of North Carolina at Chapel Hill, Chapel Hill, NC, USA

**Keywords:** triple-negative, BMI, high-fat diet, microenvironment, obese, leptin, adiponectin, genetically engineered mouse model

## Abstract

Epidemiologic studies demonstrate that obesity is associated with an aggressive subtype of breast cancer called basal-like breast cancer (BBC). Using the C3(1)-T_Ag_ murine model of BBC, we previously demonstrated that mice displayed an early onset of tumors when fed obesogenic diets in the adult window of susceptibility. Obesity was also shown to elevate mammary gland expression and activation of hepatocyte growth factor (HGF)/c-Met compared to lean controls, a pro-tumorigenic pathway associated with BBC in patients. Epidemiologic studies estimate that weight loss could prevent a large proportion of BBC. We sought to investigate whether weight loss in adulthood prior to tumor onset would protect mice from accelerated tumorigenesis observed in obese mice. Using a life-long model of obesity, C3(1)-T_Ag_ mice were weaned onto and maintained on an obesogenic high-fat diet. Obese mice displayed significant elevations in tumor progression, but not latency or burden. Tumor progression was significantly reversed when obese mice were induced to lose weight by switching to a control low-fat diet prior to tumor onset compared to mice maintained on obesogenic diet. We investigated the HGF/c-Met pathway known to regulate tumorigenesis. Importantly, HGF/c-Met expression in normal mammary glands and c-Met in tumors was elevated with obesity and was significantly reversed with weight loss. Changes in tumor growth could not be explained by measures of HGF action including phospho-AKT or phospho-S6. Other mediators associated with oncogenesis such as hyperinsulinemia and a high leptin:adiponectin ratio were elevated by obesity and reduced with weight loss. In sum, weight loss significantly blunted the obesity-responsive pro-tumorigenic HGF/c-Met pathway and improved several metabolic risk factors associated with BBC, which together may have contributed to the dramatic reversal of obesity-driven tumor progression. Future research aims to evaluate the role of obesity and the HGF/c-Met pathway in basal-like breast cancer progression.

## Introduction

Epidemiologic and other population studies suggest that obesity is a risk factor for basal-like breast cancer (BBC) – an aggressive triple-negative subtype that disproportionately affects young and African American women (1–8). Studies in various pre-clinical models of luminal sub-type breast cancers have shown that diet-induced obesity is associated with shortened mammary tumor latency (9–11), but little work has been completed on the basal-like subtype. Thus, we used a unique genetically engineered mouse model (GEMM) of BBC that most resembles human BBC (12), the C3(1)-T_Ag_ mouse model (13). We have previously demonstrated that adult-onset obesity reduced BBC latency compared to lean mice (14). Hepatocyte growth factor (HGF)/c-Met is a tumor promoting pathway that is significantly activated in BBC patient samples (15). In C3(1)-T_Ag_ mice, obesity increased HGF and its cognate receptor c-Met expression in the normal mammary gland and elevated c-Met expression and activation in tumors (14). Furthermore, our previous work has shown that BBC displays a significant relationship with stroma-secreted HGF (also known as scatter factor), a growth factor associated with tumor aggressiveness. We reported that when primary fibroblasts were isolated from mammary glands or tumors of obese mice, these fibroblasts secreted higher concentrations of HGF *ex vivo* compared to fibroblasts isolated from lean mice (14). Work from our group (15, 16) and others (17–19) reported that in humans, the HGF/c-Met signaling pathway was uniquely regulated by BBC-derived stromal cells. Interestingly, HGF is elevated in plasma of obese patients and is reduced with weight loss (20). Taken together, the HGF/c-Met pathway is one potential mechanism that is associated with obesity in mice and humans, as well as BBC samples.

Basal-like breast cancer currently has no targeted therapies (21); hence identification of modifiable risk factors would be therapeutically transformative, especially in reducing disparities associated with BBC-related mortality. Millikan et al. estimate that approximately half of BBC is attributable to obesity (4), suggesting that this subtype may be preventable through lifestyle intervention. However, it is unclear whether prevention of adiposity is needed, or whether weight loss after obesity could also be effective in reducing risk. Obesity is an epidemic in the US and worldwide (22, 23) and is one of the few important modifiable risk factors for breast cancer (24). Data on the effect of weight loss on BBC risk are limited (25–27). Hence, the intention of this study was to elucidate the effect of weight loss on BBC and the molecular mechanisms thereof. Herein, we assessed if weight reduction through dietary intervention would reverse obesity-induced BBC, and examine important metabolic parameters and the HGF/c-Met pathway. We report that when obese C3(1)-T_Ag_ mice were induced to lose weight, the diet switch group (60 → 10%) displayed significantly reduced tumor progression compared to obese mice. In addition, weight loss reversed obesity-induced HGF/c-Met expression in normal mammary gland compared to mice that remained obese. Weight loss also reduced parameters associated with metabolic syndrome including hyperinsulinemia and the leptin:adiponectin ratio. Our findings suggest that obesity-driven factors such as HGF/c-Met, insulin, and the leptin:adiponectin ratio may contribute to the onset of obesity-promoted BBCs, and that weight loss prior to tumor onset may prevent tumor progression.

## Materials and Methods

### Reagents and antibodies

Anti-mouse HGF antibody that detects total HGF (both pro and cleaved) and anti-mouse c-Met antibody that detects pro- and cleaved c-Met were obtained from R&D Systems (Minneapolis, MN, USA) (14). Adiponectin mouse ELISA kit was obtained from Abcam (ab108785; Cambridge, MA, USA). Anti-mouse pS6 (Ser235/236) (Cell Signaling 4857) and pAkt (Ser473) (Cell Signaling 3787) was obtained from Cell Signaling Technology, Inc. (Danvers, MA, USA).

### C3(1)-T_Ag_ mouse model

#### Animals and diets

**Figure 1:**
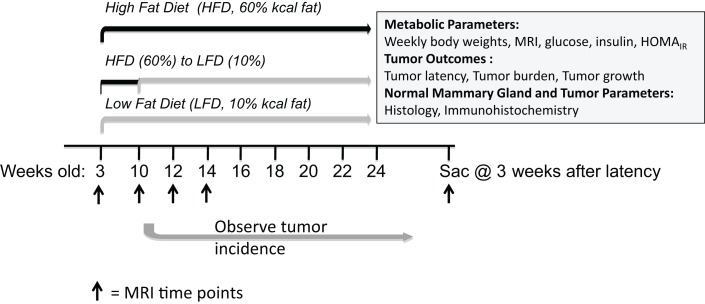
**Model of study design**. Female C3(1)-T_Ag_ mice were weaned onto control low-fat 10% kcal derived from fat (“10%”) or obesogenic 60% kcal derived from fat (“60%”) diets at 3 weeks of age. At 10 weeks of age, half of the mice on the 60% diet were switched onto the control 10% diets. Various endpoints were measured until sacrifice at 3 weeks past tumor detection.

Animal studies were performed with approval and in accordance with the guidelines of the Institutional Animal Care and Use Committee at the University of North Carolina at Chapel Hill. Female C3(1)-T_Ag_ mice were obtained in collaboration with the UNC Lineberger Comprehensive Cancer Center (LCCC) Mouse Phase I Unit (MP1U). C3(1)-Tag mice (13) were used to study the role of diet on BBC, as these mice were shown to be highly representative of human basal-like tumors (12). In females, the simian virus (SV40) large tumor antigen (Tag) is expressed in the distal mammary ductal epithelium and terminal ductal lobular unit in a hormone-independent manner leading to the development of mammary tumors in female mice (13). C3(1)-Tag mice were generated by crossing heterozygous male mice with FVB/N non-transgenic female mice. Diets obtained from Research Diets Inc. (New Brunswick, NJ, USA) were matched for protein, vitamins, and minerals, and provided 10% kcal (“10%”); and 60% kcal (“60%”) derived from fat. Details of the diet components are provided in Sundaram et al. (14). Female C3(1)-T_Ag_ weanlings were randomly assigned to various diet groups at weaning (3 weeks of age; *n* = 15 on 10% and *n* = 30 on 60%). At 10 weeks of age, half of the mice on 60% diet were switched to 10% diet (60 → 10%) (See model of study design, Figure 1).

#### Body weight and composition

Body weight was measured prior to starting mice on diet and weekly until sacrifice. Body composition including lean mass, fat mass, free water content, and total water content of non-anesthetized mice was also measured at 0, 10, 12, and 14 weeks, and at sacrifice on diet using the EchoMRI-100 quantitative magnetic resonance whole body composition analyzer (Echo Medical Systems, Houston, TX, USA). Obesity is defined as greater than a 5% incremental increase in fat composition. Fat mass is presented as percent fat mass over total body weight (14).

#### Tumor latency, number, progression (change in volume), and cell size

As described in Sundaram et al. (14) mice were monitored for tumor development by palpating three times weekly and tumor latency was defined as age at detection of first tumor. After detection of the first tumor, tumor volumes were measured weekly over 3 weeks using calipers to measure the width (short diameter) and length (long diameter) in millimeter for each tumor. The tumor volumes were calculated using the formula: length × width^2^×0.5. The percent change in volume over time (tumor progression), was calculated: (End volume – Start volume)/Start volume × 100. Tumor progression (percent change in volume) of only the first palpated tumor is presented. Percent change for *n* = 15 mice was averaged in each diet group. The total number of tumors per mouse was counted at sacrifice. Tumor cell diameter was measured using Aperio ScanScope Image Analysis Toolbox software. The longest diameters (cytoplasmic membrane; micrometer) of 30 cells from five different fields/tumor section (*n* = 150 cells) were averaged to find the overall tumor cell diameter for each sample.

#### Tissue harvest

Three weeks after detection of the first tumor, mice were anesthetized by an intraperitoneal (i.p.) injection of avertin (Fisher Scientific, Pittsburgh, PA, USA). Blood was collected by cardiac puncture into a tube with 10 μl of 0.05 mM EDTA (final conc). Plasma was separated by centrifuging blood at 500 × *g* for 5 min. Plasma was stored at −80°C. Mammary glands without palpable or visible tumors were collected as “normal,” although atypia of ductal epithelium could be present in C3(1)-T_Ag_ mice (13). Portions of the tissues were placed into a cassette and formalin fixed for immunohistochemical (IHC) analysis.

#### Measurement of metabolic parameters and plasma cytokines

Blood glucose, following a 6 h fast, was measured prior to start of diet, at diet switch, and at sacrifice following a 6 h fast using a Bayer Contour Blood Glucose Monitor (Bayer HealthCare LLC, Tarrytown, NY, USA). Metabolically relevant hormones including leptin, insulin, IL-6, MCP-1, and TNF-α were measured in the plasma collected at sacrifice using the Milliplex MAP Mouse Metabolic Hormone Magnetic Bead Panel in the Luminex MAGPIX system (EMD Millipore, Billerica, MA, USA). The homeostasis model assessment was used to calculate the approximate insulin resistance (HOMA_IR_) using the formula (blood glucose (mg/dl at sacrifice) × plasma insulin levels (at sacrifice)/405) as previously described (14, 28). Adiponectin concentrations in plasma collected at sacrifice were measured using the adiponectin mouse ELISA kit (ab108785; Abcam, Cambridge, MA, USA) following the manufacturer’s protocol. The leptin:adiponectin ratio was calculated using the measures obtained from the Luminex cytokine panel and adiponectin ELISA.

#### Immunohistochemical analyses in normal mammary glands and tumors

Immunohistochemical analysis was performed for HGF and c-Met and its downstream signals including pAkt, and pS6 following the protocol previously described in Sundaram et al. (14). Anti-mouse HGF antibody and anti-mouse c-Met antibody were used at a dilution of 1:400 with secondary donkey anti-goat antibody (1:500; Jackson Immunoresearch; # 705-065-147). Anti-mouse pS6 and pAkt antibodies were used at a dilution of 1:400 with secondary goat anti-rabbit antibody (1:500; Jackson Immunoresearch; # 111-005-003). Following staining, slides were scanned into the Aperio Scanscope CS system (Aperio Technologies, Vista, CA, USA) at a magnification of 20× and staining was quantified using the Aperio Imagescope software. The scanned slides were analyzed using the appropriate algorithms as described previously (14, 29, 30). The Aperio Imagescope software positive pixel counts for diaminobenzidine (DAB) staining in the color deconvolution algorithm was completed for HGF, pAKT, and pS6, and membrane IHC algorithm for c-Met quantification (14, 29, 30). Aperio digital analysis of DAB allows for no subjective bias in quantification. Due to our interest in the normal and tumor microenvironment, IHC (rather than Western immunoblots of total tissue lysates) and representative 40× images are presented. An *n* = 5 random areas from sections (*n* = 2 per mouse) were quantified and averaged per animal (*n* = 8 mice per diet exposure group for HGF and *n* = 5 for c-Met, pAKT, and pS6 for both normal mammary glands and tumors). Photomicrographs were obtained at a magnification of 40×.

### Statistical analysis

Data are expressed as mean ± standard error of the mean (SEM). All means were compared by one way analysis of variance (ANOVA) with Tukey’s *post hoc* test for statistical differences in SPSS (version 20) software (IBM SPSS Statistic 20.0, Armonk, NY, USA) or GraphPad Prism 5 software (GraphPad Software, Inc. La Jolla, CA, USA). Kaplan–Meier analyses were conducted using GraphPad Prism 5 software to estimate tumor latency. Log rank and chi-square tests were used to investigate differences among groups. *P* values <0.05 were considered statistically significant.

## Results

### Obesity-induced from weaning can be reversed by switching to a low-fat diet

**Figure 2:**
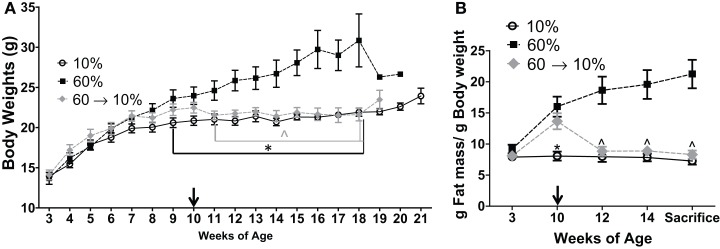
**Diet crossover induced loss of weight and fat mass compared to obese C3(1)-T_Ag_ mice**. **(A)** C3(1)-T_Ag_ mice were weighed weekly at start of diets at weaning (3 weeks of age) (*P = 0.001 from weeks 9 to end of study vs. 60%; ^∧^*P* = 0.01 from weeks 11 till sacrifice vs. 60%). **(B)** Body composition was measured by MRI at indicated weeks. Diet switch from 60% to control 10% diet is indicated with arrow in **(A,B)**. (*P < 0.05 vs. both 60 and 60 → 10%; ^∧^*P* < 0.05 vs. 60%). *n* = 15 in each diet group.

Upon weaning at 3 weeks of age, mice were fed control low-fat 10% (*n* = 15) or obesogenic 60% diets (*n* = 30). At 10 weeks of age, *n* = 15 mice on the obesogenic diet were induced to lose weight with a diet switch to 10% diet (Figure 1). Mice fed the 60% diet gained more weight than the control 10%-fed mice, and were significantly different starting at 9 weeks of age (*P* = 0.001) and remained significantly different until end of the study (Figure 2A). At week 11 (1 week post diet switch), mice on 60 → 10% diets exhibited weight loss and weights were identical to 10%-fed mice for the remainder of the study. Mice on the 60 → 10% diet weighed significantly less compared to mice on 60% diet at week 11 until the end of study (*P* = 0.01) (Figure 2A).

Mice in the 60 and 60 → 10% groups gained body fat from 3 to 10 weeks of age while on the 60% obesogenic diet, and had significantly greater body fat composition compared to the 10%-fed mice at 10 weeks of age (*P* = 0.0008, 60 → 10 vs. 10%, and *P* < 0.05, 60 vs. 10%, Figure 2B). At 12 weeks of age, 2 weeks after diet switch at 10 weeks, body fat content in the 60 → 10% mice decreased significantly to levels detected in 10%-fed mice and remained low until sacrifice. Mice fed the 60% diet exhibited greater body fat compared to 10 and 60 → 10%-fed mice from weeks 12 until sacrifice (*P* < 0.05 at weeks 12 and 14, and at sacrifice; Figure 2B). There were no significant declines in absolute lean mass in grams in any of the diet groups tested (data not shown).

### Obesity increased C3(1)-T_Ag_ tumor progression, which could be reversed by weight loss

**Figure 3:**
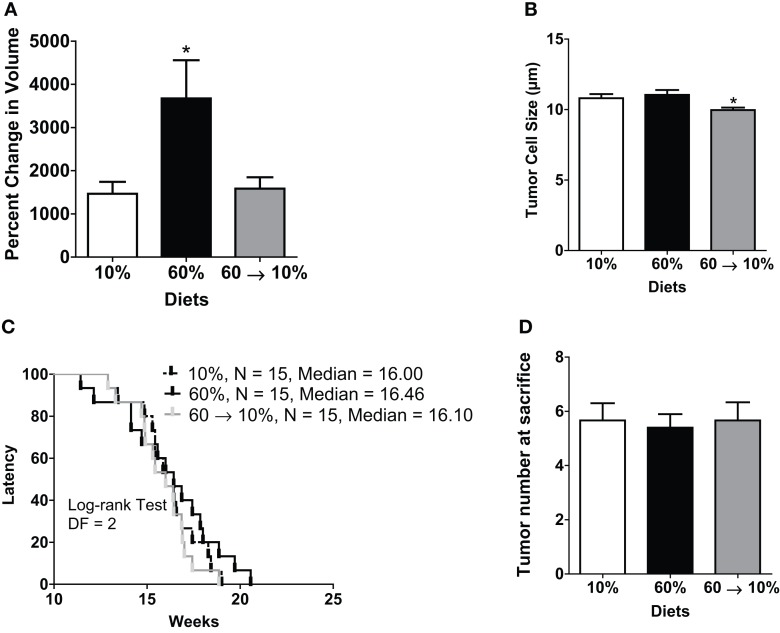
**Weight loss reduced obesity-induced tumor progression but did not change latency**. **(A)** Tumor progression measured as percent change in volume from latency to sacrifice 3 weeks later (* vs. 10% *P* = 0.001 and vs. 60 → 10% *P* = 0.0024). *n* = 15 in each diet group. **(B)** Tumor cell size was measured over five distinct fields of analyses/tumor section [* 10% (*P* = 0.043) and 60% (*P* = 0.019) vs. 60 → 10%]. *n* = 30 cells from five fields (total *n* = 150 cells) from five mice in each diet group. **(C)** Latency of first tumor identified (DF, degrees of freedom). **(D)** Total tumor burden was assessed at sacrifice. *n* = 15 in each diet group.

Tumor progression, as defined by percent change (increase) in tumor volume from time of detection over 3 weeks until sacrifice, as defined in methods, was significantly elevated in obese mice compared to lean controls (*P* = 0.001, Figure 3A). In 60 → 10%-fed mice, tumor progression was significantly inhibited compared to obese 60%-fed mice (*P* = 0.002, Figure 3A). Tumor progression in 60 → 10%-fed mice was identical to lean 10%-fed mice. Tumor progression for every tumor detected prior to sacrifice was also calculated and was identical to primary tumor (data not shown). Average tumor sizes at tumor onset (first tumor detected; latency) were identical: 22.53 ± 9.27, 22.74 ± 10.06, and 23.25 ± 9.05 mm^3^ (*P* = 0.18) in mice fed 10, 60, and 60 → 10%, respectively. In addition, there were no significant differences in tumor cell sizes measured using Aperio Scanscope Toolbar between mice on 10% diets compared to obese mice on 60% diet. However, mice fed 60 → 10% diets exhibited significantly smaller tumor cells compared with both 10% (*P* = 0.043) and 60% (*P* = 0.019) (Figure 3B). Mice on all three diets (10, 60, and 60 → 10%) had similar latencies (Figure 3C). The hazard ratios comparing 60–10% was 1.353 (95% CI of ratio: 0.63–2.9); 60 → 10–10% was 0.77 (95% CI of ratio: 0.36–1.6); and 60 → 10–60% was 0.60 (95% CI of ratio: 0.27–1.3). Mean latency in 10, 60, and 60 → 10%-fed mice was 16.15, 16.26, and 15.82 weeks, respectively. Using chi-square tests with a degree of freedom of 2, 10 vs. 60% equaled 0.60, 10 vs. 60 → 10% equaled 0.47, and 60 vs. 60 → 10% was 1.64. Tumor burden (total number of tumors) was not significantly altered by obesity or weight loss (Figure 3D).

### Obesity-induced insulin resistance and adiposity were reversible by diet intervention

**Figure 4:**
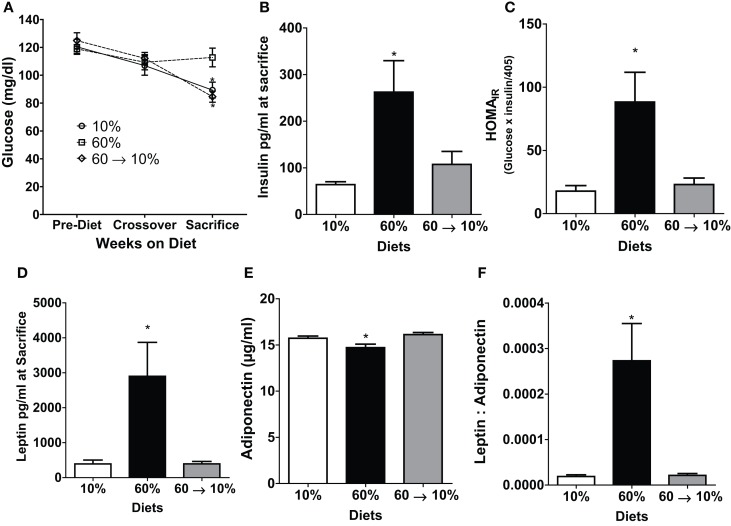
**Obesity-elevated measures of insulin resistance and adiposity were reduced by weight loss**. **(A)** 6 h fasted blood glucose was measured in weanlings pre-diet, at 10 weeks of age at diet crossover, and at sacrifice 3 weeks after tumor identification [* 10% (*P* = 0.013) and 60 → 10% (*P* = 0.0012) vs. 60%]. **(B)** Insulin was measured at sacrifice. [* 10% (*P* = 0.042) and 60 → 10% (*P* = 0.036) vs. 60%]. **(C)** HOMA_IR_ (glucose × insulin/405 was calculated from measures at sacrifice [* 10% (*P* = 0.028) and 60 → 10% (*P* = 0.013) vs. 60%]. **(D)** Leptin [* 10% (*P* = 0.0157) and 60 → 10%-fed (*P* = 0.0155) vs. 60%] and **(E)** adiponectin [* 10% (*P* = 0.0301) and 60 → 10% (*P* = 0.0032) vs. 60%] were measured at sacrifice. **(F)** Leptin: adiponectin ratios [* 10 and 60 → 10%-fed (*P* = 0.005) vs. 60%] were calculated from measures at sacrifice. *n* = 15 mice per diet group for glucose measurements and *n* = 14 for all other measures including insulin, HOMA_IR_, leptin, adiponectin, and leptin:adiponectin ratios.

We examined metabolic parameters that can contribute to obesity-induced carcinogenesis (24). Glucose levels at diet start and at diet switch were not different among any of the three groups (Figure 4A). At sacrifice, 60%-fed obese mice had significantly elevated blood glucose compared to the 10 and 60 → 10%-fed mice (*P* = 0.01 and *P* = 0.001, respectively Figure 4A). Obese mice had a 2.6-fold (*P* = 0.04) and a 2.5-fold (*P* = 0.03) elevated plasma insulin levels compared to 10 and 60 → 10%-fed animals, respectively (Figure 4B). HOMA_IR_ score, calculated as a marker of glucose intolerance, indicated that 60%-fed mice were insulin resistant compared to 10%-fed mice (*P* = 0.03). Diet switch-induced weight loss significantly blunted insulin resistance (*P* = 0.01) compared to 60%-fed mice and the levels were identical to 10%-fed mice (Figure 4C).

Leptin and adiponectin are two important adipokines associated with breast cancer (24). Leptin concentrations were significantly greater in 60%-fed mice compared to 10% (*P* = 0.02, Figure 4D). Mice on 60% diet exhibited significantly lower adiponectin levels compared to 10% (*P* = 0.03, Figure 4E). In 60 → 10%-fed mice, leptin was dramatically reduced compared to obese mice (*P* = 0.02, Figure 4D) mice. Likewise, adiponectin was elevated in 60 → 10%-fed mice compared to 60%-fed mice (Figure 4E, *P* = 0.003). There were no significant differences between 10 and 60 → 10%-fed mice in leptin or adiponectin concentrations. The leptin:adiponectin ratio is an important indicator of cancer risk (24). Leptin:adiponectin ratios of 60%-fed mice were significantly elevated compared to 10 and 60 → 10%-fed mice (*P* = 0.005; Figure 4F). Plasma levels of cytokines and chemokines associated with obesity were measured (24). No significant differences were observed in plasma concentrations of IL-6, MCP-1, or TNF-α among diet groups (Table 1).

**Table 1:** **Systemic changes in inflammatory cytokines and chemokines were not evident**.

Cytokine/chemokine	Diets		
	10%	60%	60 → 10%
IL-6	32.63 ± 8.80	35.87 ± 4.43	37.93 ± 5.23
MCP-1	45.68 ± 4.69	49.02 ± 7.84	53.98 ± 7.62
TNF-α	14.94 ± 0.95	23.80 ± 5.76	14.49 ± 1.14

*Plasma concentrations of IL-6, MCP-1, and TNF-α were measured at sacrifice for *n* = 15 mice per diet group*.

### HGF/c-Met concentrations in normal mammary glands and tumors were induced by obesity and reduced with weight loss

**Figure 5:**
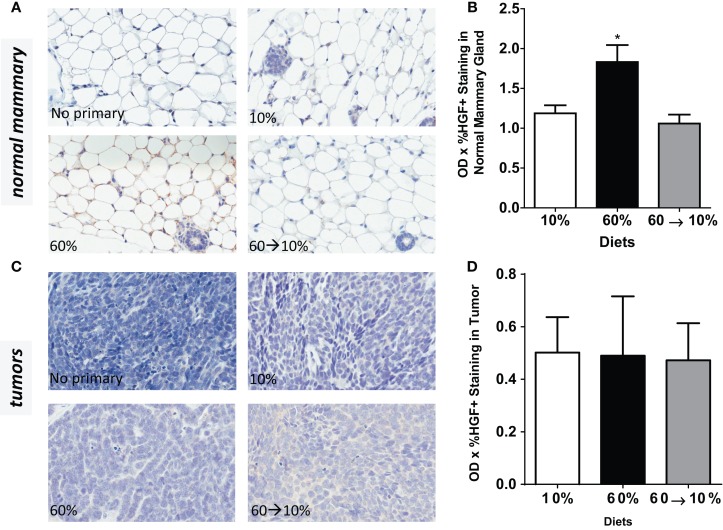
**HGF is elevated by obesity and reduced by weight loss**. **(A,B)** Representative IHC photomicrographs (40×) and HGF quantified in normal mammary gland [* 10% (*P* = 0.0102) and 60 → 10% (*P* = 0.0031) vs. 60%]. **(C,D)** Representative 40× photomicrographs and HGF quantifications in tumor. Staining was quantified in normal mammary glands and tumors in *n* = 2 sections from eight mice using Aperio Imagescope software and the color deconvolution algorithm measuring positive pixel counts for diaminobenzidine (DAB) staining. Aperio digital analysis of DAB allows for no subjective bias in quantification.

Obesity in 60%-fed mice significantly elevated HGF concentrations in the normal mammary gland (Figures 5A,B) compared to mice fed 10% diet (*P* = 0.01). Weight loss by 60 → 10% diet switch significantly reduced the HGF expression (*P* = 0.003) compared to mice fed 60% diet. HGF detected in 60 → 10%-fed mammary glands were similar to concentrations in the control 10%-fed mice (Figures 5A,B). In tumors, HGF protein concentrations were not significantly regulated by obesity or weight loss (Figures 5C,D).

**Figure 6:**
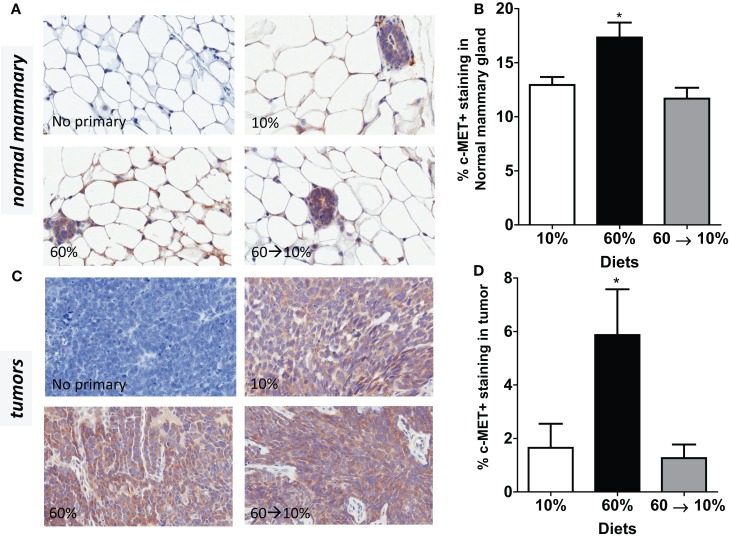
**c-Met is elevated by obesity and reduced by weight loss**. **(A,B)** Representative IHC photomicrographs (40×) and c-Met quantified in normal mammary [* 10% (*P* = 0.036) and 60 → 10% (*P* = 0.004) vs. 60%]. **(C,D)** Representative photomicrographs (40×) and tumor quantifications [* 10% (*P* = 0.043) and 60 → 10% (*P* = 0.019) vs. 60%]. Staining was quantified in normal mammary glands and tumors in *n* = 2 sections from five mice using Aperio Imagescope software and the membrane IHC algorithm for c-Met quantification.

Similarly, c-Met protein concentrations in the normal mammary gland were significantly elevated in the obese 60%-fed mice compared to 10%-fed controls (*P* = 0.04, Figures 6A,B). c-Met concentrations were significantly decreased by weight loss in the 60 → 10%-fed group compared to the obese 60%-fed mice (*P* = 0.004) and were similar to concentrations in the control 10%-fed mice (Figure 6B). In tumors, c-Met protein concentrations were also significantly elevated in the 60%-fed mice compared to the 10%-fed mice (*P* = 0.04, Figures 6C,D). Weight loss 60 → 10%-fed mice exhibited a significant decrease in c-Met protein concentrations compared to 60%-fed mice (*P* = 0.02). c-Met concentrations in 60 → 10%-fed mice were similar to levels in the lean 10%-fed mice.

### HGF-activated its downstream signal PI3K/Akt, but not p70S6K

**Figure 7:**
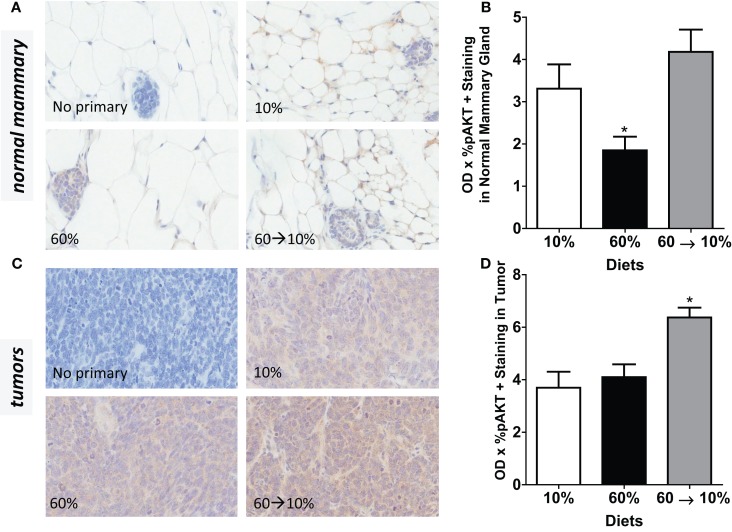
**pAkt is reduced by obesity and elevated by weight loss**. **(A,B)** Representative IHC photomicrographs (40×) and pAkt quantified in normal mammary [* 10% (*P* = 0.039) and 60 → 10% (*P* = 0.001) vs. 60%]. **(C,D)** Representative photomicrographs (40×) and tumor quantifications [* 10% (*P* = 0.001) and 60% (*P* = 0.002) vs. 60 → 10%]. Staining was quanti- fied in normal mammary glands and tumors in *n* = 2 sections from five mice using Aperio Imagescope software and the color deconvolution algorithm measuring positive pixel counts for diaminobenzidine (DAB) staining.

**Figure 8:**
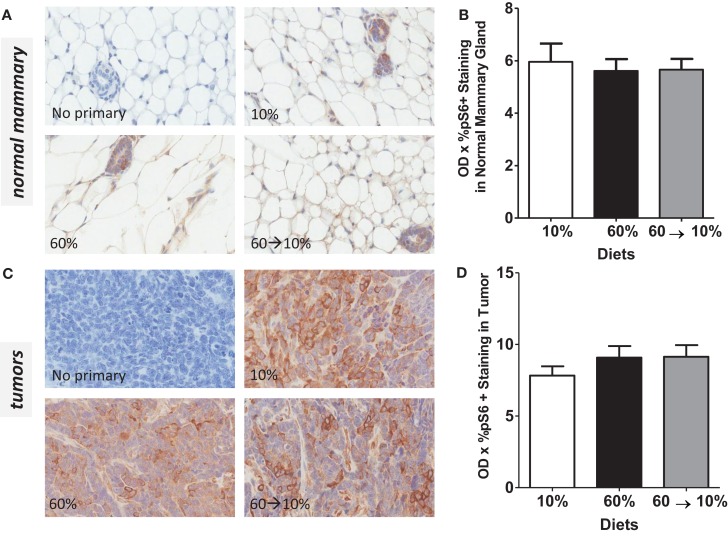
**pS6 is not modulated by obesity or weight loss**. Representative IHC photomicrographs (40×) and pS6 quantified in normal mammary **(A,B)** and tumors **(C,D)**. Staining was quantified in normal mammary glands and tumors in *n* = 2 sections from five mice using Aperio Imagescope software and the color deconvolution algorithm measuring positive pixel counts for diaminobenzidine (DAB) staining.

On HGF binding, c-Met undergoes dimerization and autophosphorylation leading to the activation of downstream phosphatidylinositol 3-kinase (PI3K)/protein kinase B (Akt) and p70 S6K signaling pathways which mediate effects of HGF including cell survival, invasion, and metastasis. We next examined HGF/c-Met signaling by measuring PI3K/Akt and p70S6K activation. Obesity significantly reduced pAkt concentrations in the normal mammary gland (Figures 7A,B) compared to mice fed 10% diet (*P* = 0.039). Weight loss by 60 → 10% diet switch significantly elevated the pAkt expression (*P* = 0.001) compared to mice fed 60% diet. Phospho-AKT concentrations in 60%-fed normal mammary glands were similar to concentrations in the control 10%-fed mice. In tumors, pAkt protein concentrations were significantly elevated by the 60 → 10% diet switch compared to mice fed 10% (*P* = 0.001) and 60% (*P* = 0.002) diets (Figures 7C,D). Phospho-S6 levels in both normal mammary glands and tumors remained unaltered by obesity or weight loss (Figures 8A–D).

## Discussion

Increases in adiposity, regardless of age, increase the risk of breast cancer (4, 5, 31–35). It has been suggested that a significant burden of BBC could be prevented by reducing obesity (4). Studies have previously reported in humans that weight loss or prevention of weight gain is protective against both pre- and post-menopausal breast cancer. Weight loss in adulthood is associated with a reduced risk of developing breast cancer compared to adult weight gain (36–39). Furthermore, women who maintained weight loss of more than 5 kg for at least 4 years after age 18 were shown to be at decreased risk of developing pre-menopausal breast cancer (40), while prevention of weight gain between age 18 years and menopause, or weight loss and maintenance of loss during these years, reduced the risk of post-menopausal breast cancer (37). Coates et al. demonstrated a statistically significant 36% reduction in risk when weight loss was achieved with respect to low-grade tumors only in pre-menopausal women (41). However, contrasting reports from three studies have shown that weight loss over a prolonged interval did not significantly reduce risk of developing breast cancer (42–44). The Women’s Health Initiative (WHI) Randomized Controlled Dietary Modification Trial in post-menopausal women indicated that a dietary intervention group exhibited 9% non-significant lower risk compared to the control group after 8 years of follow-up (45). These studies suggest that weight loss is likely beneficial in reducing risk. However, in human populations, weight loss is due to heterogeneous causes, hence, it is unclear whether dietary interventions are warranted and whether these interventions would specifically reduce BBC (27).

Studies in pre-clinical mouse models have demonstrated that diet-induced obesity is associated with shortened mammary tumor latency of mainly luminal subtype (9, 10). In BBC C3(1)-T_Ag_ GEMMs, our previous studies reported increased tumor aggressiveness as measured by significantly shortened tumor latencies after mice were made obese by high-fat diet exposure in adulthood (24). Herein, to examine if obesity-associated risk of BBC was reversible, we modeled *weight loss in adulthood* following life-long diet-induced obesity *from weaning*. Our data demonstrated that weight loss and reduction in adiposity by diet switch to a low-fat diet was achievable within a short period of time. While in our previous study, we detected obesity-shortened latency, herein when mice were exposed to diets from weaning into adulthood, there were no significant diet-induced alterations on latency or tumor burden. This demonstrates the importance of timing of administration of diet and suggests that early diet exposure in this GEMM does not alter early tumor growth (latency) since tumors were the same size when detected. However, weight loss significantly and dramatically reduced obesity-driven tumor progression to growth levels detected in lean mice. Hursting et al. have also shown that dietary energy interventions through caloric restriction suppressed progression of basal-like xenografts compared to lean control-fed mice (46). Modification of tumor growth could have occurred through changes in tumor cell size. No significant changes in cell size were detected between lean and obese, however weight loss exhibited significant decreases in tumor cell size compared to the other groups. However, when pathways known to be regulated by obesity that regulate tumor growth or tumor cell size were examined, it was evident that pAKT was regulated in the reverse manner (i.e., reduced with obesity in normal mammary). In tumors, pAkt concentrations were significantly increased with weight loss compared to both lean and obese mice. Phospho-AKT is the target of many pathways including insulin, thus activation of pAKT may be a measure of insulin sensitivity, which was reversed with weight loss to control levels. mTOR/pS6 was not regulated by obesity or weight loss in normal mammary or tumors. Thus, it was not likely that pAKT or mTOR were dramatic regulators of obesity or weight loss-mediated tumor growth in this model.

The effects of obesity on the mammary gland may depend upon the timing of exposure. Animal studies have shown that high-fat diet or obesity alters puberty onset (47, 48), mammary gland development and morphology (49–51), and tumor latency (9). Pubertal alterations including inflammatory cell composition, increased local production of growth factors, and angiogenesis may also contribute to the promotion of mammary carcinoma (52). Distinguishing between puberty and adulthood obesity-associated risks is important because both have distinct effects on stromal remodeling, and stromal-epithelial interactions modulate breast cancer behavior *in vivo* (24). During puberty, the stroma is remodeled as the gland develops to a mature, functional mammary gland (53). Once maturity is reached, changes to the stroma are less dramatic, but recent data have suggested that obesity affects mammary stroma (14, 54–57). Ongoing studies are currently investigating the effects of obesity and weight loss in pubertal versus adult windows of susceptibility to aid in clarifying when risk is most strongly associated with BBC.

Systemic or microenvironmental alterations may have contributed to tumor progression in C3(1)-T_Ag_ GEMMs. We measured metabolically regulated potential mediators such as glucose, insulin, adipokines, and inflammatory proteins. Mice made obese on the 60% diet displayed elevated glucose, insulin, HOMA_IR_ scores, and leptin and lower concentrations of adiponectin, which together are characteristics of metabolic syndrome (22, 58). Insulin is associated with greater risk of breast cancer (24, 59). In the WHI study, fasting insulin concentrations in the highest quartile were associated with 2.4-fold increase in BC risk compared to women in the lowest quartile (24). The leptin:adiponectin ratio is also an important indicator of cancer risk (24, 60). We observed increased leptin:adiponectin ratios in the obese group, which were reversed by weight loss to levels detected in lean mice. Increased obesity has been shown to correlate with increased inflammation, including inflammatory cytokines such as TNF-α, IL-6, and MCP-1 (59). However, no significant obesity- or weight-loss mediated differences in plasma levels of cytokines and chemokines were detected, suggesting that pro-inflammatory mediators likely did not contribute to tumor progression in C3(1)-T_Ag_ mice in this experimental design. Taken together, reduction in metabolically regulated hormones and adipokines, but not systemic inflammatory mediators, may have contributed to reduced tumor progression after weight loss in C3(1)-T_Ag_ mice.

Microenvironmental alterations in the normal mammary are important in BBC (61, 62). The mammary gland is similar to other adipose depots in that obesity drives stromal alterations such as elevations in immune cells or growth factors that are established contributors to breast cancer risk (14, 22, 57, 63–65). The HGF/c-Met axis is one such pathway linked to both obesity and breast cancer risk that previously had not been investigated in tandem. The HGF/c-Met signature is highly expressed in almost 90% of basal-like cancers from patients (15). HGF is elevated in obese adipose tissue, and high concentrations of serum HGF detected in obese individuals may be blunted by weight loss (20, 66). Furthermore, we previously reported elevated HGF protein concentrations in normal mammary of obese C3(1)-T_Ag_ mice compared to lean mice (14). Using *ex vivo* coculture models, we reported that proliferation and motility were specifically induced by HGF, by using blocking antibodies (14, 16). These studies suggested that HGF’s effects on proliferation and motility are likely involved in tumor progression (14, 16). Here, we report that switching C3(1)-Tag mice from obesogenic to a low-fat diet reversed HGF and c-Met expression in normal mammary glands to levels detected in lean controls. Although HGF protein concentrations in tumors were not significantly modified by obesity, similar to our previous findings (14), c-Met was dramatically elevated with obesity, and significantly blunted to control levels by weight loss. These data suggest that alterations to the HGF/c-Met pathway that occur in the normal mammary gland and tumor set the stage for tumor progression. Future studies to elucidate the efficacy of inhibiting this pathway using novel small molecule therapeutics to mitigate obesity-driven BBC need to be undertaken. Taken together, our data demonstrate that obesity increased tumor progression, which was reversed by weight loss, likely by reducing important obesity-associated metabolic and growth factors.

## Conflict of Interest Statement

The authors declare that the research was conducted in the absence of any commercial or financial relationships that could be construed as a potential conflict of interest.

## References

[1] Calle EE Rodriguez C Walker-Thurmond K Thun MJ Overweight, obesity, and mortality from cancer in a prospectively studied cohort of U.S. adults. N Engl J Med (2003) 348:1625–38 10.1056/NEJMoa021423 12711737

[2] Calle EE Kaaks R Overweight, obesity and cancer: epidemiological evidence and proposed mechanisms. Nat Rev Cancer (2004) 4:579–91 10.1038/nrc1408 15286738

[3] Carmichael AR Obesity as a risk factor for development and poor prognosis of breast cancer. BJOG (2006) 113:1160–6 10.1111/j.1471-0528.2006.01021.x 16945118

[4] Millikan RC Newman B Tse CK Moorman PG Conway K Dressler LG Epidemiology of basal-like breast cancer. Breast Cancer Res Treat (2008) 109:123–39 10.1007/s10549-007-9790-6 17578664 PMC2443103

[5] Phipps AI Malone KE Porter PL Daling JR Li CI Body size and risk of luminal, HER2-overexpressing, and triple-negative breast cancer in postmenopausal women. Cancer Epidemiol Biomarkers Prev (2008) 17:2078–86 10.1158/1055-9965.EPI-08-0206 18664548 PMC2561180

[6] Maiti B Kundranda MN Spiro TP Daw HA The association of metabolic syndrome with triple-negative breast cancer. Breast Cancer Res Treat (2010) 121:479–83 10.1007/s10549-009-0591-y 19851862

[7] Anderson GL Neuhouser ML Obesity and the risk for premenopausal and postmenopausal breast cancer. Cancer Prev Res (2012) 5:515–21 10.1158/1940-6207.CAPR-12-0091 22392012

[8] Cecchini RS Costantino JP Cauley JA Cronin WM Wickerham DL Land SR Body mass index and the risk for developing invasive breast cancer among high-risk women in NSABP P-1 and STAR breast cancer prevention trials. Cancer Prev Res (Phila) (2012) 5:583–92 10.1158/1940-6207.CAPR-11-0482 22318751 PMC4131545

[9] Gordon RR Hunter KW La Merrill M Sorensen P Threadgill DW Pomp D Genotype X diet interactions in mice predisposed to mammary cancer: II. Tumors and metastasis. Mamm Genome (2008) 19:179–89 10.1007/s00335-008-9096-y 18288525

[10] Lam JB Chow KH Xu A Lam KS Liu J Wong NS Adiponectin haploinsufficiency promotes mammary tumor development in MMTV-PyVT mice by modulation of phosphatase and tensin homolog activities. PLoS One (2009) 4:e4968 10.1371/journal.pone.0004968 19319191 PMC2656613

[11] Xu K Usary J Kousis PC Prat A Wang DY Adams JR Lunatic fringe deficiency cooperates with the Met/caveolin gene amplicon to induce basal-like breast cancer. Cancer Cell (2012) 21:626–41 10.1016/j.ccr.2012.03.041 22624713 PMC3603366

[12] Herschkowitz JI Simin K Weigman VJ Mikaelian I Usary J Hu Z Identification of conserved gene expression features between murine mammary carcinoma models and human breast tumors. Genome Biol (2007) 8:R76 10.1186/gb-2007-8-5-r76 17493263 PMC1929138

[13] Green JE Shibata MA Yoshidome K Liu ML Jorcyk C Anver MR The C3(1)/SV40 T-antigen transgenic mouse model of mammary cancer: ductal epithelial cell targeting with multistage progression to carcinoma. Oncogene (2000) 19:1020–7 10.1038/sj.onc.1203280 10713685

[14] Sundaram S Freemerman AJ Johnson AR Milner JJ Mcnaughton KK Galanko JA Role of HGF in obesity-associated tumorigenesis: C3(1)-TAg mice as a model for human basal-like breast cancer. Breast Cancer Res Treat (2013) 142:489–503 10.1007/s10549-013-2741-5 24218051 PMC3904507

[15] Casbas-Hernandez P Darcy M Roman-Perez E Brauer H Mcnaughton K Miller S Role of HGF in epithelial-stromal cell interactions during progression from benign breast disease to ductal carcinoma in situ. Breast Cancer Res (2013) 15:R82 10.1186/bcr3476 24025166 PMC3978616

[16] Brauer HA Makowski L Hoadley KA Casbas-Hernandez P Lang LJ Roman-Perez E Impact of tumor microenvironment and epithelial phenotypes on metabolism in breast cancer. Clin Cancer Res (2013) 19:571–85 10.1158/1078-0432.CCR-12-2123 23236214 PMC3684709

[17] Tuck AB Park M Sterns EE Boag A Elliott BE Coexpression of hepatocyte growth factor and receptor (Met) in human breast carcinoma. Am J Pathol (1996) 148:225–32 8546209 PMC1861613

[18] Ponzo MG Lesurf R Petkiewicz S O’Malley FP Pinnaduwage D Andrulis IL Met induces mammary tumors with diverse histologies and is associated with poor outcome and human basal breast cancer. Proc Natl Acad Sci U S A (2009) 106:12903–8 10.1073/pnas.0810402106 19617568 PMC2722321

[19] Mueller K Madden J Zoratti G Kuperwasser C List K Boerner J Fibroblast-secreted hepatocyte growth factor mediates epidermal growth factor receptor tyrosine kinase inhibitor resistance in triple-negative breast cancers through paracrine activation of Met. Breast Cancer Res (2012) 14:R104 10.1186/bcr3224 22788954 PMC3680928

[20] Bell LN Ward JL Degawa-Yamauchi M Bovenkerk JE Jones R Cacucci BM Adipose tissue production of hepatocyte growth factor contributes to elevated serum HGF in obesity. Am J Physiol Endocrinol Metab (2006) 291:E843–8 10.1152/ajpendo.00174.2006 16757549

[21] Toft DJ Cryns VL Minireview: basal-like breast cancer: from molecular profiles to targeted therapies. Mol Endocrinol (2011) 25:199–211 10.1210/me.2010-0164 20861225 PMC3035993

[22] Johnson AR Justin Milner J Makowski L The inflammation highway: metabolism accelerates inflammatory traffic in obesity. Immunol Rev (2012) 249:218–38 10.1111/j.1600-065X.2012.01151.x 22889225 PMC3422768

[23] Ogden CL Carroll MD Kit BK Flegal KM Prevalence of obesity among adults: United States, 2011–2012. NCHS Data Brief (2013) 131:1–8 24152742

[24] Sundaram S Johnson AR Makowski L Obesity, metabolism and the microenvironment: links to cancer. J Carcinog (2013) 12:19 10.4103/1477-3163.119606 24227994 PMC3816318

[25] Eliassen AH Colditz GA Rosner B Willett WC Hankinson SE Adult weight change and risk of postmenopausal breast cancer. JAMA (2006) 296:193–201 10.1001/jama.296.2.193 16835425

[26] Wolin KY Colditz GA Can weight loss prevent cancer? Br J Cancer (2008) 99:995–9 10.1038/sj.bjc.6604623 18728645 PMC2567071

[27] DeSantis C Siegel R Jemal A Breast cancer facts and figures 2013-2014. In: American Cancer Society. Atlanta, GA: American Cancer Society (2013). p. 1–38

[28] Sampey BP Vanhoose AM Winfield HM Freemerman AJ Muehlbauer MJ Fueger PT Cafeteria diet is a robust model of human metabolic syndrome with liver and adipose inflammation: comparison to high-fat diet. Obesity (Silver Spring) (2011) 19:1109–17 10.1038/oby.2011.18 21331068 PMC3130193

[29] Polcz ME Adamson LA Lu X Chang MN Fowler LJ Hobbs JA Increased IL-6 detection in adult and pediatric lymphoid tissue harboring parvovirus B19. J Clin Virol (2013) 57(3):233–8 10.1016/j.jcv.2013.02.022 23522566

[30] Ruifrok A Johnston D Quantification of histochemical staining by color deconvolution. Anal Quant Cytol Histol (2001) 23:291–299 11531144

[31] Yang XR Sherman ME Rimm DL Lissowska J Brinton LA Peplonska B Differences in risk factors for breast cancer molecular subtypes in a population-based study. Cancer Epidemiol Biomarkers Prev (2007) 16:439–43 10.1158/1055-9965.EPI-06-0806 17372238

[32] Kwan ML Kushi LH Weltzien E Maring B Kutner SE Fulton RS Epidemiology of breast cancer subtypes in two prospective cohort studies of breast cancer survivors. Breast Cancer Res (2009) 11:R31 10.1186/bcr2261 19463150 PMC2716499

[33] Stead LA Lash TL Sobieraj JE Chi DD Westrup JL Charlot M Triple-negative breast cancers are increased in black women regardless of age or body mass index. Breast Cancer Res (2009) 11:R18 10.1186/bcr2242 19320967 PMC2688946

[34] Trivers KF Lund MJ Porter PL Liff JM Flagg EW Coates RJ The epidemiology of triple-negative breast cancer, including race. Cancer Causes Control (2009) 20:1071–82 10.1007/s10552-009-9331-1 19343511 PMC4852686

[35] Mowad R Chu QD Li BD Burton GV Ampil FL Kim RH Does obesity have an effect on outcomes in triple-negative breast cancer? J Surg Res (2013) 184:253–9 10.1016/j.jss.2013.05.037 23768767

[36] Trentham-Dietz A Newcomb P Egan K Titus-Ernstoff L Baron J Storer B Weight change and risk of postmenopausal breast cancer (United States). Cancer Causes Control (2000) 11:533–42 10.1023/A:1008961931534 10880035

[37] Harvie M Howell A Vierkant RA Kumar N Cerhan JR Kelemen LE Association of gain and loss of weight before and after menopause with risk of postmenopausal breast cancer in the Iowa Women’s Health Study. Cancer Epidemiol Biomarkers Prev (2005) 14:656–61 10.1158/1055-9965.EPI-04-0001 15767346

[38] Christou NV Lieberman M Sampalis F Sampalis JS Bariatric surgery reduces cancer risk in morbidly obese patients. Surg Obes Relat Dis (2008) 4:691–5 10.1016/j.soard.2008.08.025 19026373

[39] Kawai M Minami Y Kuriyama S Kakizaki M Kakugawa Y Nishino Y Adiposity, adult weight change and breast cancer risk in postmenopausal Japanese women: the Miyagi Cohort Study. Br J Cancer (2010) 103:1443–7 10.1038/sj.bjc.6605885 20842123 PMC2990597

[40] Michels KB Terry KL Eliassen AH Hankinson SE Willett WC Adult weight change and incidence of premenopausal breast cancer. Int J Cancer (2012) 130:902–9 10.1002/ijc.26069 21413008 PMC3245343

[41] Coates RJ Uhler RJ Hall HI Potischman N Brinton LA Ballard-Barbash R Risk of breast cancer in young women in relation to body size and weight gain in adolescence and early adulthood. Br J Cancer (1999) 81:167–74 10.1038/sj.bjc.6690667 10487629 PMC2374361

[42] Ballard-Barbash R Schatzkin A Taylor PR Kahle LL Association of change in body mass with breast cancer. Cancer Res (1990) 50:2152–5 2317807

[43] Brinton LA Swanson CA Height and weight at various ages and risk of breast cancer. Ann Epidemiol (1992) 2:597–609 10.1016/1047-2797(92)90004-A 1342311

[44] Trentham-Dietz A Newcomb PA Storer BE Longnecker MP Baron J Greenberg ER Body size and risk of breast cancer. Am J Epidemiol (1997) 145:1011–9 10.1093/oxfordjournals.aje.a009057 9169910

[45] Prentice RL Caan B Chlebowski RT Patterson R Kuller LH Ockene JK Low-fat dietary pattern and risk of invasive breast cancer: the women’s health initiative randomized controlled dietary modification trial. JAMA (2006) 295:629–42 10.1001/jama.295.6.629 16467232

[46] Dunlap SM Chiao LJ Nogueira L Usary J Perou CM Varticovski L Dietary energy balance modulates epithelial-to-mesenchymal transition and tumor progression in murine claudin-low and basal-like mammary tumor models. Cancer Prev Res (2012) 5:930–42 10.1158/1940-6207.CAPR-12-0034 22588949 PMC3822442

[47] Hoversland RC Onset of obesity and puberty in genetically obese SHHF/Mcc-cp rats. Int J Obes Relat Metab Disord (1992) 16:977–84 1335977

[48] Brill DS Moenter SM Androgen receptor antagonism and an insulin sensitizer block the advancement of vaginal opening by high-fat diet in mice. Biol Reprod (2009) 81:1093–8 10.1095/biolreprod.109.079301 19605781 PMC2802232

[49] La Merrill M Kuruvilla BS Pomp D Birnbaum LS Threadgill DW Dietary fat alters body composition, mammary development, and cytochrome p450 induction after maternal TCDD exposure in DBA/2J mice with low-responsive aryl hydrocarbon receptors. Environ Health Perspect (2009) 117:1414–9 10.1289/ehp.0800530 19750107 PMC2737019

[50] Olson LK Tan Y Zhao Y Aupperlee MD Haslam SZ Pubertal exposure to high fat diet causes mouse strain-dependent alterations in mammary gland development and estrogen responsiveness. Int J Obes (Lond) (2010) 34:1415–26 10.1038/ijo.2010.51 20231845 PMC2923244

[51] Hue-Beauvais C Chavatte-Palmer P Aujean E Dahirel M Laigre P Pechoux C An obesogenic diet started before puberty leads to abnormal mammary gland development during pregnancy in the rabbit. Dev Dyn (2011) 240:347–56 10.1002/dvdy.22536 21246651

[52] Zhao Y Tan YS Aupperlee MD Langohr IM Kirk EL Troester MA Pubertal high fat diet: effects on mammary cancer development. Breast Cancer Res (2013) 15:R100 10.1186/bcr3561 24156623 PMC3978633

[53] Sternlicht M Key stages in mammary gland development: the cues that regulate ductal branching morphogenesis. Breast Cancer Res (2006) 8:201 10.1186/bcr1368 16524451 PMC1413974

[54] Adler HI The use of microbial membranes to achieve anaerobiosis. Crit Rev Biotechnol (1990) 10:119–27 10.3109/07388559009068263 2202519

[55] Le TT Rehrer CW Huff TB Nichols MB Camarillo IG Ji-Xin C Nonlinear optical imaging to evaluate the impact of obesity on mammary gland and tumor stroma. Mol Imaging (2007) 6(3):205–11 17532886 PMC2653856

[56] Morris PG Hudis CA Giri D Morrow M Falcone DJ Zhou XK Inflammation and increased aromatase expression occur in the breast tissue of obese women with breast cancer. Cancer Prev Res (2011) 4:1021–9 10.1158/1940-6207.CAPR-11-0110 21622727 PMC3131426

[57] Sun X Casbas-Hernandez P Bigelow C Makowski L Joseph Jerry D Smith Schneider S Normal breast tissue of obese women is enriched for macrophage markers and macrophage-associated gene expression. Breast Cancer Res Treat (2012) 131:1003–12 10.1007/s10549-011-1789-3 22002519 PMC3640411

[58] Hursting SD Hursting MJ Growth signals, inflammation, and vascular perturbations: mechanistic links between obesity, metabolic syndrome, and cancer. Arterioscler Thromb Vasc Biol (2012) 32:1766–70 10.1161/ATVBAHA.111.241927 22815342

[59] Hursting SD Digiovanni J Dannenberg AJ Azrad M Leroith D Demark-Wahnefried W Obesity, energy balance, and cancer: new opportunities for prevention. Cancer Prev Res (2012) 5:1260–72 10.1158/1940-6207.CAPR-12-0140 23034147 PMC3641761

[60] Rogozina OP Bonorden MJ Seppanen CN Grande JP Cleary MP Effect of chronic and intermittent calorie restriction on serum adiponectin and leptin and mammary tumorigenesis. Cancer Prev Res (Phila) (2011) 4:568–81 10.1158/1940-6207.CAPR-10-0140 21257708 PMC3071428

[61] Troester MA Lee MH Carter M Fan C Cowan DW Perez ER Activation of host wound responses in breast cancer microenvironment. Clin Cancer Res (2009) 15:7020–8 10.1158/1078-0432.CCR-09-1126 19887484 PMC2783932

[62] Camp JT Elloumi F Roman-Perez E Rein J Stewart DA Harrell JC Interactions with fibroblasts are distinct in basal-like and luminal breast cancers. Mol Cancer Res (2011) 9:3–13 10.1158/1541-7786.MCR-10-0372 21131600 PMC3045848

[63] Bissell MJ Radisky D Putting tumours in context. Nat Rev Cancer (2001) 1:46–54 10.1038/35094059 11900251 PMC2975572

[64] Casbas-Hernandez P Fleming JM Troester M Gene expression analysis of in vitro cocultures to study interactions between breast epithelium and stroma. J Biomed Biotechnol (2011) 2011:520987 10.1155/2011/520987 22203785 PMC3238808

[65] Sun X Gierach GL Sandhu R Williams T Midkiff BR Lissowska J Relationship of mammographic density and gene expression: analysis of normal breast tissue surrounding breast cancer. Clin Cancer Res (2013) 19:4972–82 10.1158/1078-0432.CCR-13-0029 23918601 PMC4073678

[66] Swierczynski J Korczynska J Goyke E Adrych K Raczynska S Sledzinski Z Serum hepatocyte growth factor concentration in obese women decreases after vertical banded gastroplasty. Obes Surg (2005) 15:803–8 10.1381/0960892054222678 15978151

